# Genomic landscape in Saudi patients with hepatocellular carcinoma using whole-genome sequencing: a pilot study

**DOI:** 10.3389/fgstr.2023.1205415

**Published:** 2023-08-04

**Authors:** Mazen Hassanain, Yang Liu, Weam Hussain, Albandri Binowayn, Duna Barakeh, Ebtehal Alsolme, Faisal AlSaif, Ghaida Almasaad, Mohammed AlSwayyed, Maram Alaqel, Rana Aljunidel, Sherin Abdelrahman, Charlotte A. E. Hauser, Saleh Alqahtani, Robert Hoehndorf, Malak Abedalthagafi

**Affiliations:** ^1^ Department of Surgery, Faculty of Medicine, King Saud University, Riyadh, Saudi Arabia; ^2^ Computational Bioscience Research Center, King Abdullah University for Science and Technology, Thuwal, Saudi Arabia; ^3^ Genomic Research Department, King Fahad Medical City, Riyadh, Saudi Arabia; ^4^ Department of Pathology, King Khalid University Hospital (KKUH), King Saud University (KSU), Riyadh, Saudi Arabia; ^5^ Laboratory for Nanomedicine, Division of Biological and Environmental Science and Engineering (BESE), King Abdullah University of Science and Technology (KAUST), Thuwal, Saudi Arabia; ^6^ KAUST Smart Health Initiative (KSHI), King Abdullah University of Science and Technology (KAUST), Thuwal, Saudi Arabia; ^7^ Computational Bioscience Research Center (CBRC), King Abdullah University of Science and Technology (KAUST), Thuwal, Saudi Arabia; ^8^ Red Sea Research Center (RSRC), King Abdullah University of Science and Technology (KAUST), Thuwal, Saudi Arabia; ^9^ Center for Outcomes Research in Liver Diseases, Washington, DC, United States; ^10^ Division of Gastroenterology and Hepatology, Johns Hopkins University, Baltimore, MD, United States; ^11^ Liver Transplant Center, King Faisal Specialist Hospital and Research Center, Riyadh, Saudi Arabia; ^12^ Pathology and Laboratory Medicine Department, Emory School of Medicine, Emory University, Atlanta, GA, United States

**Keywords:** HCC, WGS, NGS, Saudi, sorafenib resistance

## Abstract

**Background and aims:**

Hepatocellular carcinoma (HCC) is the third most prevalent cancer in Saudi Arabia. HCC poses a significant clinical challenge due to the presence of resistance among certain patients to the standard therapeutic agent sorafenib. This study aims to unravel the genomic characteristics of HCC patients in Saudi Arabia, investigate the genetic makeup of tumors in both sorafenib-sensitive and sorafenib-resistant patients, and analyze the functional implications of genomic abnormalities observed in these individuals. The resistance displayed by some HCC patients toward sorafenib underscores the need for alternative treatment approaches to effectively combat this formidable disease burden.

**Methods:**

Whole-genome sequencing (WGS) was performed on 16 HCC samples and targeted sequencing was performed on seven additional tumors. We identified and validated somatic and germline genetic aberrations. Employing a prize-collecting Steiner tree algorithm, we identified important altered genetic modules and potential biomarkers for each patient. Furthermore, we analyzed non-synonymous germline and somatic mutations, specifically in patients who underwent sorafenib treatment.

**Results:**

Out of the 13 patients who received sorafenib, three exhibited sorafenib sensitivity, while the others showed resistance to the drug. Notably, 3 out of 16 individuals carried cancer-predisposing mutations. Additionally, 8 out of 16 patients displayed non-synonymous somatic alterations in genes associated with cancer. In the targeted-sequencing samples, rare non-synonymous variants were observed across all seven cases. The study also revealed the presence of specific somatic aberrations, including *TP53*, *PIK3CA*, *APOB*, *CTNNB1*, *DPYD*, *LRP1B*, *MYC*, and *NFE2L2*, which were identified in two patients. Among the 42 genes linked to sorafenib treatment, 4 out of 10 resistant patients carried somatic non-synonymous variants. Furthermore, when analyzing the 5,000 genes most relevant to the 42 genes, 7 out of 10 resistant individuals exhibited rare non-synonymous germline variants. Interestingly, none of the three sorafenib-sensitive patients displayed any concerning variants in those genes.

**Conclusion:**

Our findings indicate that most of the HCC patients possess cancer-related genetic variants, and the altered pathways in these patients exhibit similarities. Notably, resistant patients exhibit a higher frequency of aberrations in sorafenib-related genes than do sensitive patients. Specifically, 4 out of 10 resistant individuals demonstrated 13 somatic mutations, whereas none of the three sensitive patients exhibited any. Similarly, 7 out of 10 resistant patients possessed 30 germline mutations, while none were observed in the sensitive group (two-sided Fisher’s exact test; somatic: *p*=0.50, germline: 0.07). These results contribute to our understanding of the genetic landscape of HCC and highlight potential therapeutic targets that could aid in overcoming treatment resistance.

## 1 Introduction

Liver cancer is ranked the third most common cancer in Saudi Arabia and the sixth most common cancer worldwide. Hepatocellular carcinoma (HCC) is the most common type of primary liver cancer. It is considered a prototypical inflammation-associated cancer, and, hence, it has a number of risk factors including obesity, alcohol, and hepatitis virus infection with underlying cirrhosis. However, 25% of HCCs result in a non-cirrhotic liver (1). Treatment of HCC is usually employed as a regimen of therapies including targeted therapies, as it is known to be resistant to conventional chemotherapy, especially in patients who have depleted hepatic reserves and cirrhosis. Since the vascular endothelial growth factor receptor (VEGFR) pathway is involved in the pathogenesis of HCC, sorafenib is an oral multikinase inhibitor with potent effects against VEGFR receptors (2). Reported variants associated with the VEGFR pathway include p.Trp88Arg, p.Leu163Pro, and p.His191Asp on the *VHL* gene, p.Val600Glu on the *BRAF* gene, p.Thr315Ile on the *ABL1* gene, p.Arg217Ser on the *MYOF* gene, and p.Arg22Ter on the *SDHD* gene (3). Since DNA polymorphism and mutations are common in cancer, HCC can also develop resistance when mutations involve the Wnt/β-catenin pathway, oncogenes, and tumor-suppressor genes. Identifying these mutations is essential to further optimize the treatment regimen (4). This study aimed to identify the genomic characteristics of Saudi patients with HCC, the genomic makeup of the tumors between sorafenib-sensitive and sorafenib-resistant patients, and to analyze the functional consequences of genomic aberrations that occurred in the studied patients.

## 2 Methods, sample collection, DNA extraction, and processing

Discovery samples were fresh–frozen biopsies. DNA from 16 fresh–frozen samples was extracted using the ZR-Duet DNA/RNA MiniPrep Plus kit (Zymo Research, Irvine, CA) in accordance with the manufacturer’s instructions. Seven validation samples were FFPE (formalin fixed paraffin embedded) tumor blocks with a tumor percentage of >90%. DNA from FFPE samples was manually extracted from the blocks using the GeneRead™ DNA FFPE Kit (QIAGEN, Germantown, MD, USA). We designed a custom targeted sequencing panel that focused on the most frequently altered genes in HCC and the commonly altered genes from the discovery cohort WGS by using Ion Ampliseq Designer (Thermo Fisher Scientific). The panel covers all exons of 66 protein-coding genes (5–7).

### 2.1 Patients and ethical approval

Discovery samples (tumor and matched blood) were prospectively collected fresh from 16 Saudi patients diagnosed in the period 2012–2017 from King Saud University (KSU), and an additional seven samples were collected for validation from FFPE cases at King Faisal Specialist Hospital and Research Center (KFSHRC) Riyadh. All samples were histologically diagnosed as HCC. We declare that informed consent was obtained from all participants in adherence with the Declaration of Helsinki and Research Advisory Committees (RAC) rules and regulations under the following approved project at KSU and KFSHRC. All protocols were carried out in accordance with relevant guidelines and regulations. Participants gave informed consent to participate in the study before taking part. All statistical analyses were conducted using R statistical software v4.1.2 and R package XNomial (function”xmulti”) v1.0.4.

#### 2.1.1 Sequencing and variant calling

Sixteen fresh–frozen tissue samples underwent WGS using a NovaSeq 6000 S4 flow cell; the read length was 150 bases, the depth was around 50x–60x and the kit used was the NEBnext Ultra II DNA library prep kit following the manufacturer’s protocol. In brief, DNA ranging from 500 pg to 1 μg was fragmented and used in the subsequent steps. End repair was then performed on the DNA fragments to facilitate subsequent adaptor ligation. Following the end repair, adaptor ligation was done. This was followed by size selection and cleanup of the adaptor-ligated DNA. Next, PCR enrichment was performed to amplify the DNA fragments with attached adaptors, allowing for their subsequent analysis and sequencing. To ensure the quality and size distribution of the enriched DNA, a cleanup of the PCR reaction was conducted. Following the cleanup step, the size distribution of the DNA was checked using an Agilent Bioanalyzer High Sensitivity DNA chip. The libraries were then loaded on an S4 flow cell for sequencing.

We used Bcbio-nextgen (https://github.com/chapmanb/bcbio-nextgen) (8) for tumor-normal calling with mutect2 (for somatic variant), as well as GATK-haplotype (for germline variant). Finally, we annotated the variants using several databases (3, 9, 10). FastQC mean quality scores for all 16 samples were more than 30. The alignment rates were more than 99.2%. The average read coverage was around 36 for normal tissue samples and 28 for tumor samples. The average number of germline variation SNPs and germline insertions/deletions were around 4.5 million and 0.6 million, respectively. The average number of somatic number varients (SNPs) and somatic insertions/deletions are around 0.2 million and 13,000, respectively.

Library preparation for the HCC custom panel was performed using the Ion AmpliSeq library kit version 2.0 (ThermoFisher Scientific) in accordance with the manufacturer’s guidelines. Pooled libraries were loaded onto the Ion 530 Chip (Thermo Fisher Scientific) and processed in the Ion Chef Instrument (ThermoFisher Scientific). Sequencing was performed on the Ion S5 XL system (Thermo Fisher Scientific).

### 2.2 Genetic analysis

The cancer genome interpreter (CGI) is a platform that annotates the potential of alterations detected in tumors to act as drivers and their possible effects on treatment response (11).

We downloaded a catalog of validated oncogenic mutations and selected germline variants predisposed to cancer. Our custom-designed HCC-targeted panel included all genes of interest and other HCC genes of interest in The Cancer Genome Atlas Program (TCGA) HCC cohort.

We focused only on rare non-synonymous variants:

(1) gnomAD AF (frequency of existing variants in gnomAD exomes combined population) less than 0.01.
(2) protein-truncating variants that have a high impact in the consequence (including stop gained, frameshift variant, splice acceptor variant, splice donor variant, and start loss) and missense variants.

### 2.3 Differential gene analysis

We used TCGAbiolinks to perform a gene differential analysis (12). We downloaded liver hepatocellular carcinoma gene expression data from TCGA project (13), which involves 424 samples and 19,947 genes. We obtained 714 significant genes (logFC > 2, false discovery rate (FDR)< 0.01), which served as prize nodes in the next step.

### 2.4 Prize-collecting Steiner tree algorithm

We use the protein–protein interaction (PPI) network for human proteins downloaded on 29 April 2020 from the STRING database version 11.0 (14). We removed interactions with a confidence score of less than 700. The remaining interaction network consisted of 17,182 proteins with 841,069 interactions.

Many studies have suggested prize-collecting Steiner tree (PCST) algorithms as potential methods to identify cancer driver genes (15) and cancer-related signaling pathways (16). A PCST algorithm was demonstrated for two breast cancer signatures (17).

We used a PCST algorithm to identify the most important module for each individual. PCST tries to find a connected subnetwork integrating as many interested genes as possible (18):


(1) $$o\left(F\right)\;=\;\beta \sum_{v\notin V}\;p\left(v\right)\;+\;\sum_{v\in E}\;c\left(e\right)$$

V is a gene, and the prize is the division of the number of non-synonymous variants in this gene-by-gene length. E is the edge between two genes, and the cost is one minus the interaction confidence score percentage. β is a parameter that controls the tradeoff between including prizes and excluding expensive edges.

We then used PPI as prior knowledge to propagate information and detect important disease modules for each individual. We used a PCST algorithm for these 16 individuals to detect the most important module.

### 2.5 Network guilt-by-association using random walk with restart

We downloaded genetic mutations of 42 genes in PHARMGKB that related to sorafenib (19).

We then applied the network guilt-by-association analysis to the PPI network. Given the 42 genes as bait genes, we used the random walk with restart to calculate association scores for each gene with the bait genes. In a network with *n* nodes, the random walk with restart was defined as (20):


(2) $$\;p_{t+1\;}=\left(1-\gamma \right)Wp_{t\;}+\;\gamma p_{0}$$

where $p_{0}$ is the initial probability vector in which equal probabilities are assigned to the starting nodes; $p_{t\;}$ is the probability of the vector containing the probabilities of the nodes at step t; γ is the restarting probability; and W is a column-normalized adjacency matrix of the network. Genes with higher association scores are more functionally associated with sorafenib-related genes.

### 2.6 Sorafenib resistance analysis

We analyzed genetic mutations of 42 genes in PHARMGKB that related to sorafenib. We had a total of 13 patients who received sorafenib treatment. Three of them were sensitive to the drug and are still alive. Another 10 of them raised drug resistance and died. We evaluated both non-synonymous somatic variants and rare non-synonymous germline variants and compared between sensitive and resistant patients.

## 3 Results

### 3.1 Clinical data

We collected clinical data for all 16 patients and the findings are summarized in **Table 1**. The number of female patients and male patients was equivalent, with a mean age of 64.8 years (SD = 16.61 years). The body mass index (BMI) of the patients ranged between 16.99 and 42.73. Two patients were infected with hepatitis B virus (HBV) and six patients were infected with hepatitis C virus (HCV). We also highlight the patients who were administered with sorafenib treatment in **Table 1**.

**Table 1:** Clinical data for 16 HCC patients.

**Gender**	Male: 8 (16) – 50% Female: 8 (16) – 50%	
**Age**	31–92 years old Mean: 65.1 years	
**BMI**	16.99–42.73 Mean: 27.6	
**Hepatitis**	HBV	HCV
	Positive: 2 (16) – 12.5%	Positive: 6 (16) – 37.5%
**Sorafenib treatment**	Yes	No
	13(16) – 82.3%	3 (16) – 17.6%

### 3.2 Genetic analysis from 16 WGS samples

We compared the validated predisposition variants from CGI with our germline variants for 16 patients, and two of them could be detected: rs152451 was present in three individuals (heterozygotes were in case 1 and case 6, and homozygotes were in case 14), and rs17217772 was present in one individual (heterozygote was in case 1).

**Figure 1:**
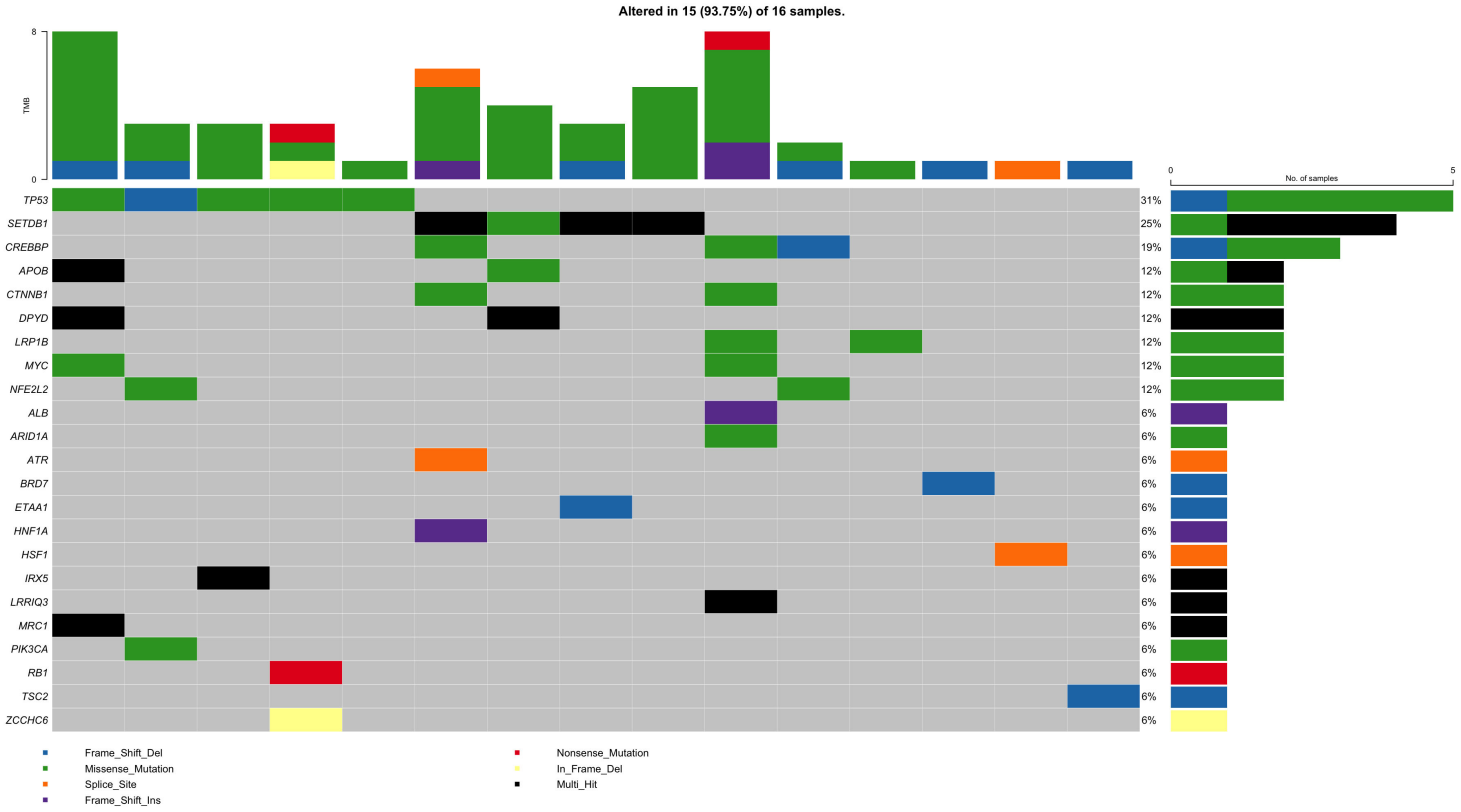
The somatic mutational landscape of 16 HCC patients on genes of interest. Each row represents a gene and each column represents a patient. The right bar plot represents the gene mutational status among 16 HCC patients. The above bar plot represents the tumor mutation burden in each patient.

We also analyzed rare non-synonymous somatic variants on 23 genes of interest, and 15 of 16 patients could be detected as having somatic mutations in the genes in **Figure 1**.

We integrated the results to see a combined germline–somatic landscape in HCC (**Table 2**), and 11 of 16 individuals carried either a predisposition variant or rare non-synonymous somatic variants. One carried a predisposition homozygous variant and two carried compound heterozygotes somatic variants.

**Table 2:** Number of germline/somatic variants on genes of interest in 11 HCC patients.

Patients code	No.com_hete	No.homo	No.hete
**1 (LS1P0001-V01-LIV1)**	0	0	2
**3 (LS1P0002-V02-LIV1)**	0	0	1
**4 (LS1P0006-V01-LIV1)**	0	0	5
**5 (LS1P0023-V02-LIV1)**	0	0	3
**6 (LS1P0008-V01-LIV1)**	0	0	1
**9 (LS1P0014-V01-LIV1)**	0	0	1
**10 (LS1P0015-V01-LIV1)**	2	0	3
**11 (LS1P0025-V01-LIV1)**	0	0	2
**12 (LS1P0026-V01-LIV1)**	0	0	2
**14 (LS1P0034-V01-LIV1)**	0	1	0
**16 (LS1P0024-V01-LIV)**	1	0	5

Eleven out of 16 individuals carried either a predisposition variant or rare non-synonymous somatic variants. com_hete represents compound heterozygotes, homo represents homozygote, and hete represents heterozygote.

We then tested if those seven validated individuals have any of the rare non-synonymous variants in the gene panel. All of the seven samples carried a rare non-synonymous variant in *BRD7* and five individuals carried a non-synonymous variant in APOB. We also tested these genes in the TCGA cohort and found that the *CREBBP* (logFC = 5.04, FDR = 1.77e-46), *XRCC6* (logFC = 2.08, FDR = 1.49e-27), and *ETAA1* (logFC = –2.98, FDR = 3.46e-48) genes are differentially expressed.

### 3.3 Identify important PPI module and genes in HCC

Via 16 PCST trees, we obtained 437 genes and 572 interactions. In these 437 genes, 5 were included in the gene panel (*TP53*, *PIK3CA*, *ETAA1*, *CTNNB1*, *CREBBP*), and 39 were validated on DisGeNet with evidence (21). Other frequent genes such as *CD44* and nuclear factor-B (*NF-B*) were also shown to be relevant to HCC (22, 23). Among these modules, the KRT18 module, DENND3 module, AKR1C1 module, and ADAM8 module were found to be frequently detected (altered) in patients. KRT18 was shown to be relevant to cryptogenic cirrhosis and cirrhosis, familial (24). DENND3 circRNAs were also found to be upregulated in HCC (25). Studies also showed that hepatitis B virus X protein up-regulates AKR1C1 expression through nuclear factor-Y in HCC (26). High ADAM8 expression was also shown to be associated with poor prognosis in patients with HCC (27).

### 3.4 Sorafenib resistance analysis

There are no mutations of any of the related genes in the sensitive group. However, there are three individuals who obtained concerned mutations in the resistant group: cases 5, 10, 11, and 16 (**Table 3**).

**Table 3:** Number of somatic mutations on sorafenib-related genes on 13 sorafenib-treated HCC patients.

Gene	Sensitive			Resistant									
	2	3	7	4	5	6	8	9	10	11	12	15	16
**SLC15A2**	0	0	0	0	0	0	0	0	0	0	0	0	1
**RET**	0	0	0	0	0	0	0	0	1	0	0	0	0
**FLT1**	0	0	0	0	0	0	0	0	1	0	0	0	0
**NOS3**	0	0	0	0	0	0	0	0	1	0	0	0	0
**SLC22A1**	0	0	0	0	0	0	0	0	1	0	0	0	0
**ABCG2**	0	0	0	0	1	0	0	0	0	0	0	0	1
**FLT4**	0	0	0	0	0	0	0	0	0	0	0	0	1
**CYP3A4**	0	0	0	0	0	0	0	0	0	0	0	0	1
**CYP2C8**	0	0	0	0	0	0	0	0	1	1	0	0	1
**ABCC2**	0	0	0	0	1	0	0	0	0	0	0	0	1
**HIF1A**	0	0	0	0	0	0	0	0	0	0	0	0	1
**CYP2B6**	0	0	0	0	0	0	0	0	1	0	0	0	1
**MAPK12**	0	0	0	0	0	0	0	0	0	0	0	0	1
**RAF1**	0	0	0	0	1	0	0	0	0	0	0	0	0
**ABCB1**	0	0	0	0	1	0	0	0	1	0	0	0	0
**MAPK4**	0	0	0	0	1	0	0	0	0	0	0	0	0
**SLCO1B1**	0	0	0	0	1	0	0	0	1	0	0	0	1
**Total**	0	0	0	0	6	0	0	0	8	1	0	0	10

We then measured how germline variants could contribute to sorafenib resistance (**Table 4**). We applied the network guilt-by-association method to identify more associated genes with those 42 genes and selected the top 5,000 genes as candidate genes. There are no mutations of any of the related genes in the sensitive group. However, six out of eight individuals in the resistant group carried at least one rare non-synonymous variant in those 5,000 genes (occurring on only nine genes): cases 11, 12, 15, 16, 8, 4, and 5. Among these nine genes, six are included in the Drug–Gene Interaction Database (28).

**Table 4:** Number of germline mutations on sorafenib-related genes on 13 sorafenib-treated HCC patients.

Gene	Sensitive			Resistant									
	2	3	7	4	5	6	8	9	10	11	12	15	16
***BARD1***	0	0	0	0	1	0	0	0	0	1	0	0	0
***SDHC***	0	0	0	0	0	0	0	0	0	0	1	0	0
***NF1***	0	0	0	0	0	0	1	0	0	0	1	0	0
***MLH1***	0	0	0	0	0	0	0	0	0	0	0	1	0
***MET***	0	0	0	0	1	0	0	0	0	0	0	1	0
***XPC***	0	0	0	1	0	0	0	0	0	0	0	0	1
***FANCC***	0	0	0	0	0	0	0	0	0	0	0	0	1
***SDHAF2***	0	0	0	0	0	0	1	0	0	0	0	0	0
***CDH1***	0	0	0	0	0	0	1	0	0	0	0	0	0
**Total**	0	0	0	1	2	0	3	0	0	1	2	2	2

## 4 Discussion

The carcinogenesis of HCC involves a complicated yet gradual process of alterations that accumulate to formulate the neoplastic cells. Being mostly resistant to conventional chemotherapy, research is currently investing more in precision medicine (29)

Most Saudi HCC patients carried either germline or somatic non-synonymous variants in cancer-related genes. The PCST algorithm revealed important modules for each patient. Many genes in these modules were validated to be relevant to HCC; other genes frequently shared between most patients were also shown to be contributing to HCC. The network guilt-by-association method identified more HCC-related genes and showed that these related genes were more frequently mutated in the resistant group than in the sensitive group.

Most known HCC driver genes were detected to be mutated somatically in patients. For example, *TP53*, *PIK3CA*, *APOB*, *CTNNB1*, *DPYD*, *LRP1B*, *MYC*, and *NFE2L2* were identified to mutate in at least two patients. Eleven out of 16 individuals carried either a predisposition variant (rs152451 and rs17217772) or rare non-synonymous somatic variants. One carried a predisposition homozygous variant and two carried compound heterozygotes somatic variants.

The PCST algorithm identified important altered modules for each patient. In the shared gene pools identified in most patients, 39 HCC-related genes were found, such as *TP53* and *CTNNB1*. Other genes were also shown to potentially contribute to HCC. For example, *KRT8* and *KRT18* were detected in 15 and 16 individuals, respectively. Studies have shown that a high keratin 8/18 ratio predicts an aggressive hepatocellular cancer phenotype (13). *ADAM8* was also screened out in all 16 individuals. Researchers illustrate that expression levels of the metalloproteinase *ADAM8* critically regulate proliferation, migration, and malignant signaling events in hepatoma cells (14).

The sorafenib-related genes were more likely to be inherited and mutated in the resistant group than in the sensitive group. Comparing between two groups that gained mutations in 42 sorafenib genes, only 4 patients in the resistant group had mutations. By evaluating the germline variant mutation status in the associated genes, only seven individuals in the resistant group carried rare non-synonymous variants. Among those genes, *NF1*, *BARD1*, *XPC*, *MET*, *FANCC*, and *CDH1* are included in the Drug–Gene Interaction Database.

However, our study has some limitations. The discovery sample size is small due to a lack of biobanking facilities in the KSA for cancer patients. Additionally, while we collected over 40 cases for validation from multiple centers, only seven cases passed the QC. With the sample size limitation, we could not subgroup the patients and evaluate the subtype influence.

In conclusion, our study comprehensively analyzed the effect of mutations on related genes on HCC from both inherited and gained aspects in the Saudi population. The HCC panel was a cost-effective strategy for mutation screening in routine diagnostic HCC samples. PCST and the guilt-by-association network method also enabled us to identify more genes contributing to HCC and sorafenib resistance.

## Data availability statement

The datasets presented in this article are not readily available because of the restriction in the Saudi Arabia law. Requests to access the datasets should be directed to the corresponding author and after getting approval from the NBEC https://ncbe.kacst.edu.sa/en/.

## Ethics statement

Discovery samples were prospectively collected from 16 Saudi patients at one local tertiary center diagnosed in the period of 2012–2017 from King Saud University, additional 7 cases were collected for validation from King Faisal Specialist Hospital and Research Center-Riyadh. IRB approval was obtained from all participating centers. The patients/participants provided their written informed consent to participate in this study.

## Author contributions

MH, WH, GA, DB, FA, MaA, MoA, RA, and SAl collected samples for discovery and validation. AB, SAb, and EA performed the genetics experiments. YL performed variant calling and annotation for 16 WGS samples and downstream analysis. YL, MAb, and DB wrote the initial manuscript. RH, and CH edited the manuscript. MAb designed the study, oversaw it, and extensively edited the manuscript. All authors contributed to the article and approved the submitted version.
