# Supplementary material for: Genomic landscape in Saudi patients with hepatocellular carcinoma using whole-genome sequencing: a pilot study
Source: Front Gastroenterol (Lausanne). 2023 Aug 4;2:1205415. doi: 10.3389/fgstr.2023.1205415 (PMC12952451; doi:10.3389/fgstr.2023.1205415)
Supplement: Supplementary file 1 [file DataSheet_1.pdf]

germline nonsynonymous variant on sorafenib related genes
Patient ID
11	BARD1:rs61754118
12	SDHC:rs200375156	NF1:rs17887014
15	MLH1:rs4647256	MET:rs761243391
16	XPC:rs577415509	FANCC:rs730881724
4	XPC:rs768979551
5	BARD1:rs61754118	MET:rs761243391
8	SDHAF2:rs111402137	CDH1:rs33935154	NF1:rs17887014

Patient ID
somatic nonsynonymous variant on sorafenib genes
10	SLC22A1:rs12208357	ABCB1:rs2032582	NOS3:rs540435018
	RET:rs1799939	CYP2C8:rs2071426	SLCO1B1:rs4149056
	FLT1:rs35549791	CYP2B6:rs35303484
11	CYP2C8:.
16	SLC15A2:rs1143672	ABCG2:rs2231137	FLT4:rs146806202
	CYP3A4:rs148317879	CYP2C8:rs11572080
ABCC2:rs8187710	SLCO1B1:rs769900186	HIF1A:rs41508050
CYP2B6:rs8192709	MAPK12:rs1129880
5	RAF1:.	ABCG2:rs2231137	ABCB1:rs2032582	ABCC2:rs2273697
SLCO1B1:rs2306283	MAPK4:rs3752087
